# Downregulation of RBM17 enhances cisplatin sensitivity and inhibits cell invasion in human hypopharyngeal cancer cells

**DOI:** 10.1515/med-2023-0669

**Published:** 2023-03-15

**Authors:** Xiaolin Wang, Deshang Chen, Guoying Han, Xiaomin Wang, Xuebao Liu, Binbin Xu, Weiwei Liu, Hui Li, Mingjie Zhang, Shiyin Ma, Yuefeng Han

**Affiliations:** Department Of Otolaryngology Head and Neck Surgery, The First Affiliated Hospital of Bengbu Medical College, Bengbu, Anhui 233000, China; Department Of Otolaryngology Head and Neck Surgery, The First Affiliated Hospital of Bengbu Medical College, 287 Changhuai Road, Bengbu, Anhui 233000, China

**Keywords:** hypopharyngeal squamous cell carcinoma, RNA-binding motif protein 17, epithelial–mesenchymal transition, cisplatin

## Abstract

Most of advanced hypopharyngeal squamous cell carcinoma (HSCC) are resistant to chemotherapy, and there is still lack of effective treatment for HSCC now. The present study aimed to investigate whether downregulation of RNA-binding motif protein 17 (RBM17) could enhance cisplatin sensitivity and inhibit cell invasion in HSCC and the underlying mechanism. We observed that RBM17 was upregulated in tumor tissues and associated with poor progression. Treatment of FaDu cells with cisplatin increased RBM17 expression in mRNA levels. Downregulation of RBM17 enhanced cisplatin-mediated inhibition of FaDu cells. In addition, downregulation of RBM17 effectively suppressed tumor cell migration and invasion through the reversion of epithelial–mesenchymal transition. Moreover, downregulation of RBM17 could significantly slow tumor growth in FaDu xenograft tumor model. Liquid chromatography–mass spectrometry/mass spectrometry detection and independent PRM analysis showed that 21 differentially expressed proteins were associated with the downregulation of RBM17. Taken together, our study implied that downregulation of RBM17 could serve as a novel approach to enhance cisplatin sensitivity in HSCC.

## 1 Introduction

Hypopharyngeal squamous cell carcinoma (HSCC) is the poorest prognosis tumor of head and neck squamous cell carcinoma (HNSCC), which arise from the mucosa of the hypopharynx and account for about 5% of all head and neck cancers [1]. Due to there is no obvious symptoms in early stages, most of patients with HSCC were diagnosed as at stage III or IV. Although huge progress had been made for treating HSCC, which contains primary surgery with pre- or postoperative radiotherapy and primary radiotherapy with chemotherapy, the 5-year overall survival (OS) with advanced HSCC is lower than 40% [2]. Cisplatin-based chemotherapy plays important role in the treatment of HNSCC. Disappointedly, HSCC is resistant to chemotherapy [3]. Therefore, it is urgently need to elucidate the mechanism of chemoresistance and the development of new therapeutic approaches.

Previous studies revealed that alternative pre-mRNA splicing is a vital molecular mechanism for promoting proteomics diversity, which plays a crucial role in tumor progress [4]. Both *cis*-regulatory elements, i.e., short RNA sequence motifs, and *trans*-acting factors, i.e., RNA-binding proteins, play essential role in the regulation of alternative splicing [5,6,7]. RNA-binding motif protein 17 (RBM17), also named splicing factor 45, was first identified as a member of the spliceosome [8]. RBM17 harbors an unstructured N-terminal domain, an a-helical G-patch motif involved in protein–protein and protein–nucleic acid interactions and a C-terminal RRM domain required for mRNA splicing [9]. In terms of tumor research, RBM17 can promote the proliferation of tumor cells in liver cancer cells and is associated with poor prognosis, so it can be used as a potential therapeutic target for liver cancer [10]. RBM17 binds to tiRNA-Gly and induces alternative splicing of thyroid carcinoma to promote cell proliferation and migration [11]. In addition, it is reported that overexpression of RBM17 can induce multidrug resistance to anticancer drugs. In our previous studies, it has been demonstrated that knockdown of RBM17 can inhibit the proliferation of FaDu cells in hypopharyngeal cancer [12]. However, no study has reported the effects of RBM17 on drug resistance, cell migration, and invasion in hypopharyngeal cancer.

Therefore, RBM17 in hypopharyngeal carcinoma was selected as the research object in this study. Through targeted proteomics, FaDu xenograft model, and other experimental methods, the aim is to explore the effect and molecular mechanism of downregulation of RBM17 to improve cisplatin sensitivity and inhibit hypopharyngeal cancer, so as to provide a basis for elucidating the mechanism of cisplatin resistance in hypopharyngeal cancer and discovering new potential therapeutic targets.

## 2 Materials and methods

### 2.1 Patient samples and cell line

HSCC tissues and adjacent normal tissues were collected from six patients, who were hospitalized in The First Affiliated Hospital of Bengbu Medical College from 2021 to 2022. FaDu cells were purchased from BeNa Culture Collection Incorporation Co., Ltd., and maintained at 37°C in a humidified 5% CO_2_ atmosphere in Dulbecco’s modified Eagle’s medium supplemented with 10% fetal bovine serum (FBS), 100 IU/ml penicillin, and 100 µg/ml streptomycin (Hyclone, GE).


**Ethics approval and consent to participate:** The present human and animal studies were approved by the Ethics Committee of The First Affiliated Hospital of Bengbu Medical College, and patients provided written informed consent.
**Informed consent:** Informed consent has been obtained from all individuals included in this study.

### 2.2 Lentiviral construction for shRNA treatment

RBM17-targeted shRNA (5′-ACTTAAGTGTCCTACTAAA-3′, GenBank NM_032905) and the scramble negative control sequence (5′-AATTCTCCGAACGTGTCACGT-3′) were cloned into the pGV115-green fluorescent protein lentiviral vector (Shanghai Genechem Co., Ltd., Shanghai, China).

### 2.3 Cell proliferation assays

Cell proliferation was evaluated by cell counting kit-8 (CCK-8) assay. In brief, FaDu cells were seeded in a 96-well plate at 5,000 cells per well and cultured in 37°C incubator for 24 h. After treatment with different concentrations of cisplatin for 24 h, 100 μl of CCK-8 solution was added to each well. Two hours later, absorbance was measured at a wavelength of 450 nm with a plate reader.

### 2.4 Wound healing assays

Twelve-well plate was seeded with LV-scramble or LV-shRBM17-infected FaDu cells at a density of 100,000 cells/well. The monolayer cells were scratched by a sterile pipette tip and washed three times by phosphate-buffered saline (PBS). Photographic images were taken at 0, 24, and 48 h.

### 2.5 Cell migration and invasion assays

The migration and invasion abilities of FaDu cells were investigated by using a 24-well Transwell chamber (diameter 6.5 mm, 8 μm pore size; Corning, Corning, NY, USA). Briefly, the infected FaDu cells were resuspended in serum-free medium and seeded in the upper chamber at a density of 3 × 10^4^ cells/well. The medium with 10% FBS was added to the bottom chamber. For invasion assays, the upper chamber was pre-treated with Matrigel (50 μl; BD Biosciences). After incubation in a 5% CO_2_ humidified incubator at 37°C for 24 h, the non-migrating cells were scraped with cotton swabs and the migrated and invaded cells were fixed with 4% paraformaldehyde and then stained with 5% crystal violet. Cells were quantified in three random fields using an optical inverted microscope at a magnification of 200×.

### 2.6 Liquid chromatography–mass spectrometry (LC–MS)

The tryptic peptides were dissolved in 0.1% formic acid (solvent A), and then separated using the EASY-nLC 1000 ultra-high performance liquid system. The peptides are separated by the ultra-high-performance liquid system and injected into the NSI ion source for ionization and then analyzed by a Q Exactive mass spectrometer (Thermo Fisher Scientific). The mass spectrometer was operated in a positive ion mode. Mass spectrometry (MS) data were acquired using a data-dependent top 10 method dynamically choosing the most abundant precursor ions from the survey scan (350–1,800 *m*/*z*) for HCD fragmentation. Survey scans were acquired at a resolution of 70,000 at *m*/*z* 200 with an AGC target of 3e6 and a maxIT of 50 ms. MS2 scans were acquired at a resolution of 17,500 for HCD spectra at *m*/*z* 200 with an AGC target of 2e5 and a maxIT of 45 ms, and isolation width was 2 *m*/*z*. Only ions with a charge state between 2 and 6 and a minimum intensity of 2e3 were selected for fragmentation. Dynamic exclusion for selected ions was 30 s. Normalized collision energy was 30 eV. MS/MS raw files were processed using MASCOT engine (Matrix Science, London, UK; version 2.6) embedded into Proteome Discoverer 2.2 and searched against the Uniprot_HomoSapiens_20367_20200226 database. Except for TMT labels, carbamidomethyl (C) was set as a fixed modification. Variable modifications were oxidation (M) and acetyl (protein N-term). A peptide and protein false discovery rate of 1% were enforced using a reverse database search strategy. Proteins with fold change >1.2 and *P* value (Student’s *t* test) <0.05 were considered to be differentially expressed proteins [13,14].

### 2.7 qRT-PCR

Total RNA was extracted from tissue and FaDu cells using the TRIzol reagent (Invitrogen). Complementary DNA (cDNA) was reverse transcribed by the PrimeScriptÔ 1st Strand cDNA Synthesis Kit (Takara, Kusatsu, Japan) according to the manufacturer’s instructions. The SYBRR Premix Ex TaqÔ (TaKaRa) was used for qRT-PCR. The2^−ΔΔCt^ method was adopted and applied to calculate the relative expression. mRNA was normalized using GADPH, which were reported in our previous study [15]. The list of primers is shown in Table 1.

**Table 1:** Primer sequences used for qRT-PCR

Gene	Primer sequence (5′-3′)
*E-cadherin*	F: GAAGTGTCCGAGGACTTTGG
	R: CAGTGTCTCTCCAAATCCGATA
*Vimentin*	F: TGTCCAAATCGATGTGGATGTTTC
	R: TTGTACCATTCTTCTGCCTCCTG
*ZEB1*	F: GCACAACCAAGTGCAGAAGA
	R: GCCTGGTTCAGGAGAAGATG
*RBM17*	F: AGTGGAGACCAGTGACTCAAA
	R: CTGGGGCGAGGACTGTACT
*GAPDH*	F: CAGCCTCAAGATCATCAGCA
	R: TGTGGTCATGAGTCCTTCCA

### 2.8 Western blot analysis

FaDu cells and tissue were collected and resuspended in cell lysis buffer for western blot on ice. BCA protein quantification kit was used to detect the protein concentration. Protein from each sample was loaded onto an SDS-PAGE and transferred onto polyvinylidene difluoride membranes (Millipore, Temecula, CA, USA). Membranes were blocked with 5% skim milk in phosphate-buffered saline containing 0.1% Tween-20 (PBS-T). To detect the target proteins, membranes were incubated with primary antibodies Vimentin (1:2000, Proteintech Group), E-cadherin (1:5000, Proteintech Group), GAPDH (1:3000, Proteintech Group), ZEB1 (1:500, Proteintech Group), ARHGDIB (1:5,000; Abcam), UHRF1 (1:500; Abcam), CCN1 (1:1,000; Abcam) and β-actin (1:2,000; Abcam) overnight at 4°C. Membranes were then washed for 10 min with PBS-T for three times and incubated with secondary antibody (1:2,000; Abcam) for 2 h at room temperature. The membranes were washed and developed using chemiluminescence reagents (Millipore) and visualized with gel imaging equipment (Bio-Rad, USA).

### 2.9 *In vivo* tumorigenesis

The experiments were performed with the approval of Animal Care and Committee of Bengbu Medical College. Six-week-old BALB/c female nude mice were purchased from Beijing Vital River Animal Company. A number of 1 × 10^7^ logarithmically growing FaDu cells (LV-scramble and LV-shRBM17) were subcutaneously inoculated into the left flank of mice. The diameters of the tumors were measured every 2 days, and the tumor volumes were calculated.

### 2.10 Bioinformatic analysis

The “Expression analysis-Box Plots” module was used to get box plots of the expression difference between these tumor tissues and the corresponding normal tissues of the GTEx (Genotype-Tissue Expression) database and “Survival Analysis” module was applied to obtain OS and disease-free survival (DFS) data with 50% cutoff by online tool of Gene Expression Profiling Interactive Analysis (GEPIA) (http://gepia.cancer-pku.cn) database [16]. We used the UALCAN portal to analyze cancer omics data and conduct protein expression analysis of the clinical proteomic tumor analysis consortium (CPTAC) dataset [17].

### 2.11 Statistical analysis

Data were analyzed using GraphPad Prism 8.0 software (GraphPad Software Inc., CA) and were expressed as mean ± standard error of mean. Difference among the groups was determined using analysis of variance or unpaired *t* test. All experiments were repeated at least three times. *P* < 0.05 was considered to be statistically significant.

## 3 Results

### 3.1 Gene expression profiling of RBM17 in normal human versus tumor tissues

**Figure 1:**
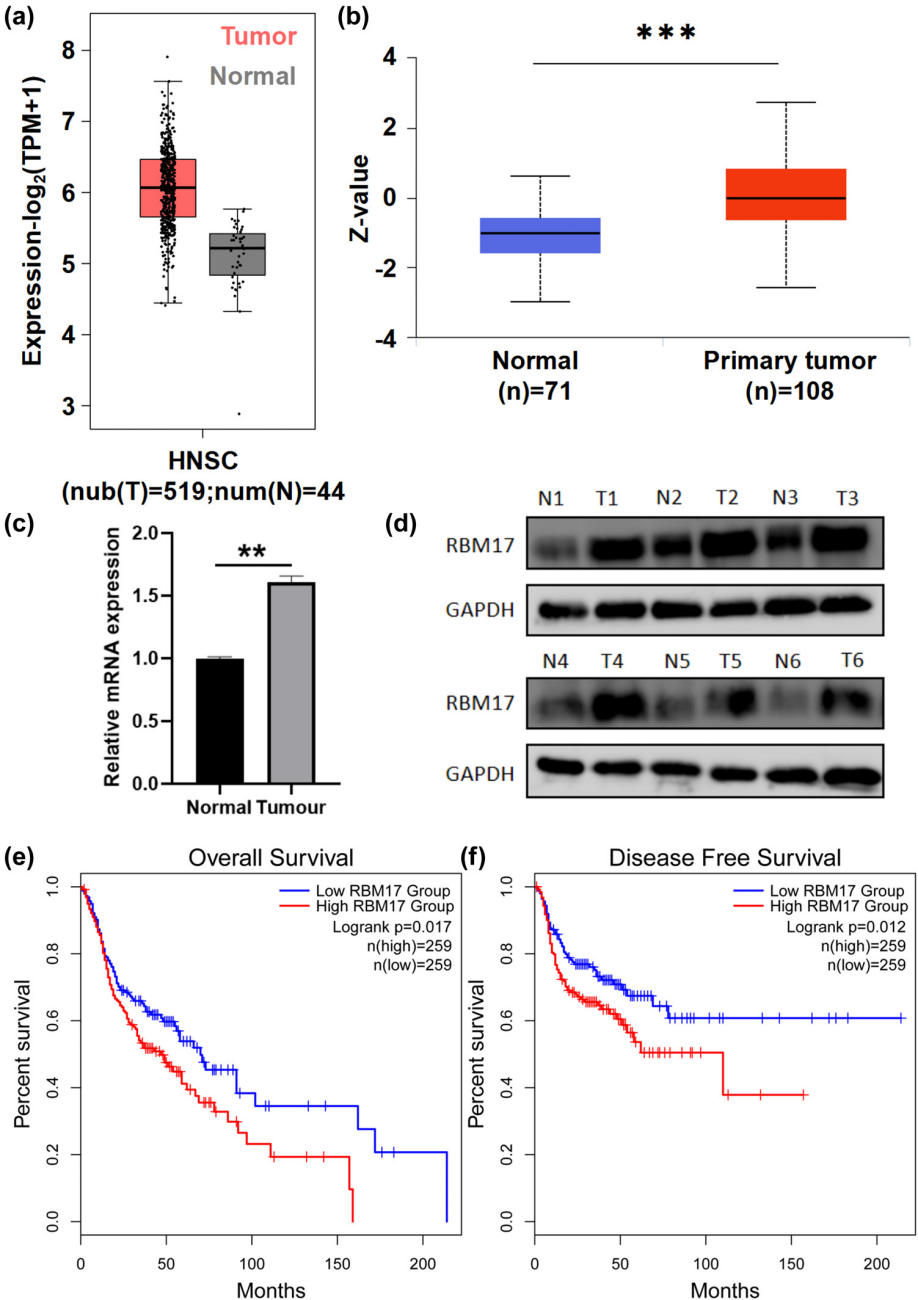
RBM17 is upregulated and associated with a poorer prognosis in HNSCC. (a) The expression of RBM17 in the TCGA database was compared with the expression of the corresponding normal tissues in the GTEx database, which was analyzed by GEPIA. (b) The protein level of RBM17 in primary tumor tissues and normal tissues from CPTAC database. (c and d) Expression level of RBM17 was confirmed by qRT-PCR and western blot in HSCC tissues and normal tissues. (e and f) Kaplan–Meier survival curve analysis of RBM17 for the OS and DFS in HNSCC from GEPIA database. **P < 0.01, ***P < 0.001.

First, we applied the TCGA and GTEx datasets to evaluate the expression of *RBM17* in tumor tissues and normal tissues by the GEPIA. As shown in Figure 1a, compared to normal tissues, *RBM17* transcript levels were significantly increased in tumor tissues. In addition, the CPTAC dataset displayed increasing level of RBM17 protein in the primary tumor tissues (Figure 1b). In addition, qRT-PCR and western blot results confirmed that RBM17 expression was upregulated in HSCC tissues compared with adjacent normal tissues (Figure 1c and d). Kaplan–Meier survival curves from the GEPIA database demonstrated that high expression of RBM17 was significantly associated with shorter OS time and DFS time (*P* = 0.017; *P* = 0.012) (Figure 1e and f).

### 3.2 Downregulation of RBM17 inhibits HSCC proliferation and sensitizes HSCC to cisplatin

**Figure 2:**
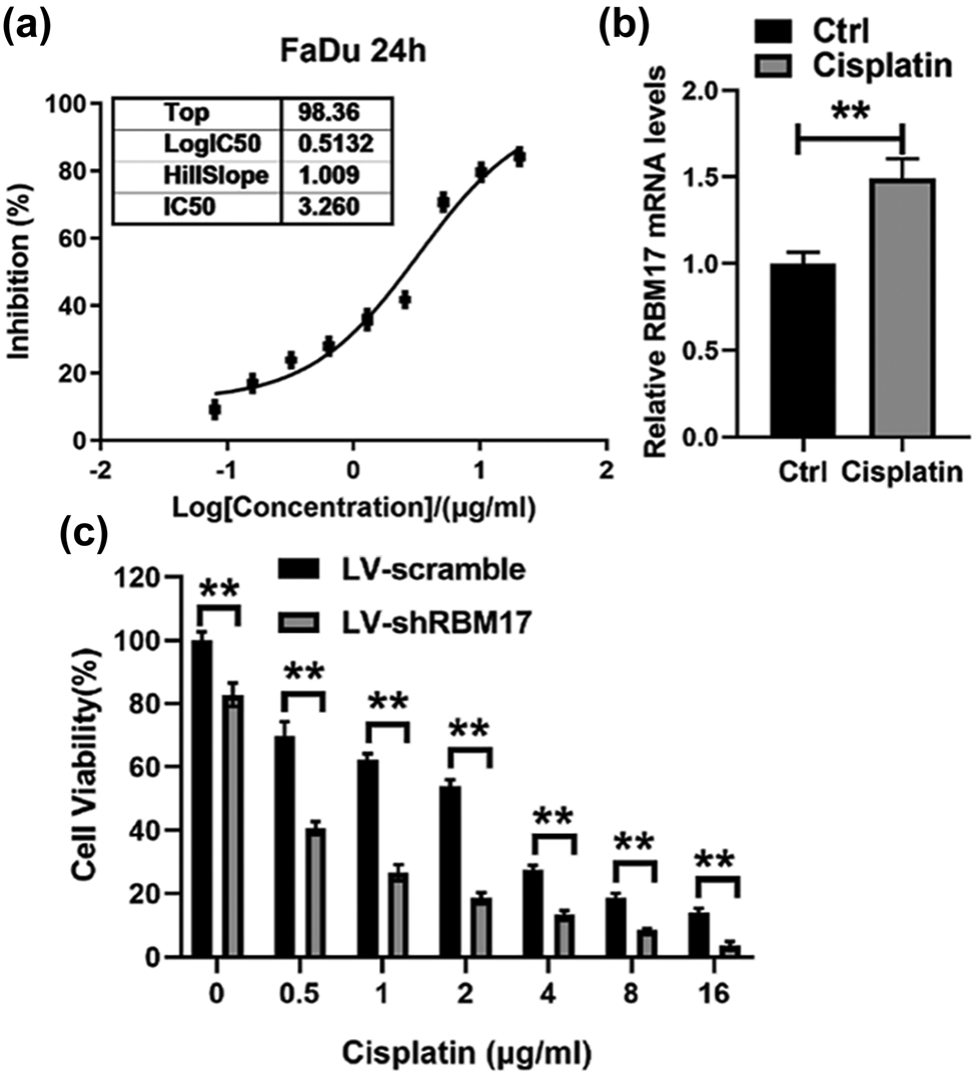
Downregulation of RBM17 enhances cisplatin sensitivity *in vitro*. (a) Inhibition of cisplatin on FaDu cells was in a concentration-dependent manner (b) FaDu cells were treated with 3.26 μg/ml cisplatin for 24 h, and the mRNA level of RBM17 was detected by real-time PCR. (c) LV-shRBM17- or LV-scramble-infected cells were incubated with different concentrations of cisplatin for 24 h, and the cell viability was determined by CCK-8 assays. **P < 0.01.

To investigate the effect of cisplatin on the growth and proliferation of HSCC, FaDu cells were treated with gradient concentration of cisplatin. Cisplatin significantly decreases the proliferation of FaDu cells; the IC_50_ value is about 3.26 μg/ml (Figure 2a). Furthermore, we examined whether cisplatin can regulate the expression of RBM17. Interestingly, real-time PCR result indicated that cisplatin can increase the RBM17 mRNA level (Figure 2b). The effect of RBM17 knockdown on the sensitivity of FaDu cells to cisplatin was then evaluated by CCK-8 assays. The results showed that downregulation of RBM17 by lentiviral vector significantly decreases cell viability of cisplatin-treated FaDu cells (Figure 2c).

### 3.3 Downregulation of RBM17 inhibits the invasion, migration, and epithelial–mesenchymal transition (EMT) processes of FaDu cells

**Figure 3:**
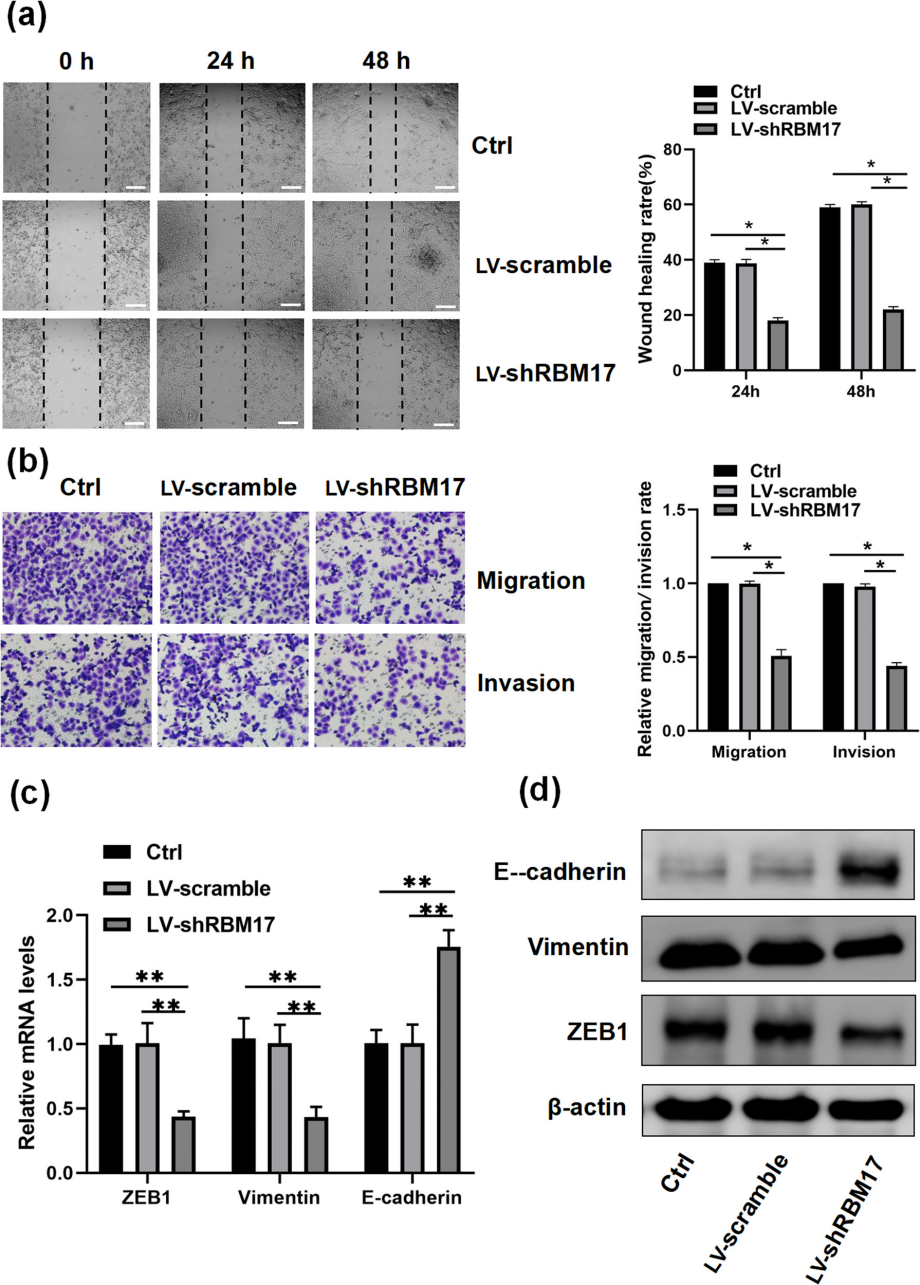
Knockdown of RBM17 affects FaDu cell invasion, migration, and EMT. (a) Cell migration ability was examined by the wound-healing assay. Images were taken using a 4× objective lens. (b) The cell invasion ability was investigated using a Transwell assay. (c and d) ZEB1, Vimentin, and E-cadherin expressions in LV-shRBM17 and LV-scramble cells were detected by qRT-PCR and western blot. *P < 0.05, **P < 0.01.

Our previous study demonstrated that the acquisition of cisplatin resistance is related with a more aggressive and invasive phenotype in nasopharyngeal carcinoma [15]. To assess whether knockdown RBM17 can affect the migration and invasion ability of FaDu cells, we performed wound-healing and Transwell assays. We found that cell migration and invasion were remarkably decreased in LV-shRBM17 cells compared with LV-scramble cells (Figure 3a and b). In these results, we deduced that the RBM17 pathway may induce EMT in HSCC. We detected mRNA and protein expression of EMT-related genes. Protein and mRNA levels of EMT markers Vimentin and transcript factor ZEB1 were significantly decreased, but the expression level of epithelial marker E-cadherin was remarkably increased in LV-shRBM17 cells, compared with LV-scramble cells (Figure 3c and d).

### 3.4 Downregulation of RBM17 decreases tumor growth in an animal model

**Figure 4:**
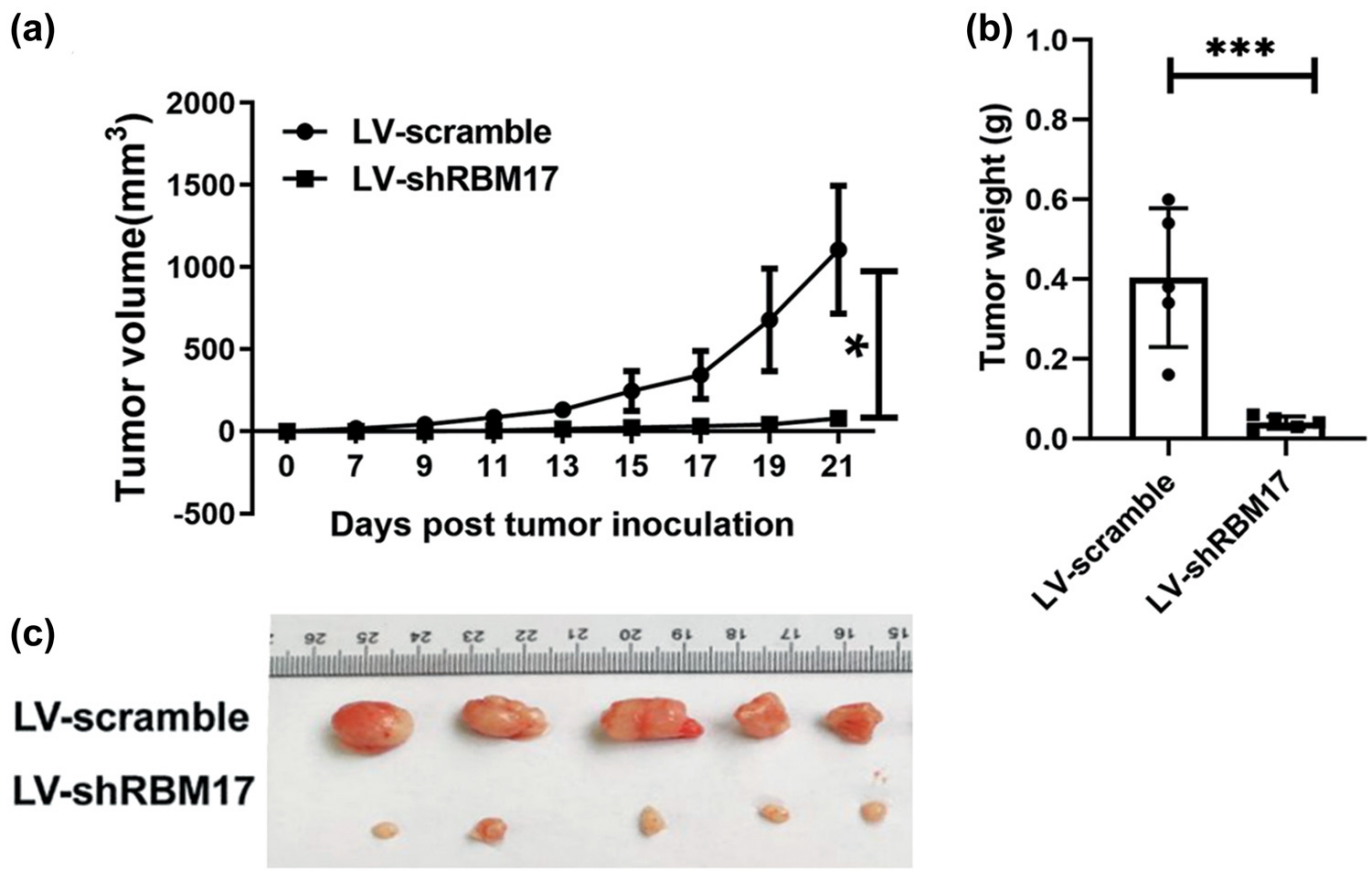
Knockdown RBM17 inhibits FaDu HSCC tumor growth *in vivo*. (a) Tumor volume was measured every 2 days from day 7 to day 21. (b) Tumor weight was calculated at day 21. (c) Representative images of FaDu HSCC xenograft tissues in different groups. *P < 0.05, ***P < 0.001.

We evaluated the effect of RMB17 downregulation on the HSCC growth in a xenograft mice model. Flank tumors were established in nude mice inoculated with FaDu cells stably expressing shRNA directed against the RBM17. Downregulation of RBM17 significantly inhibited tumor growth (Figure 4a) and reduced tumor burden (Figure 4b and c), relative to the control mice.

### 3.5 Analysis of differentially expressed proteins of RBM17-downregulated cells vs control cells

**Figure 5:**
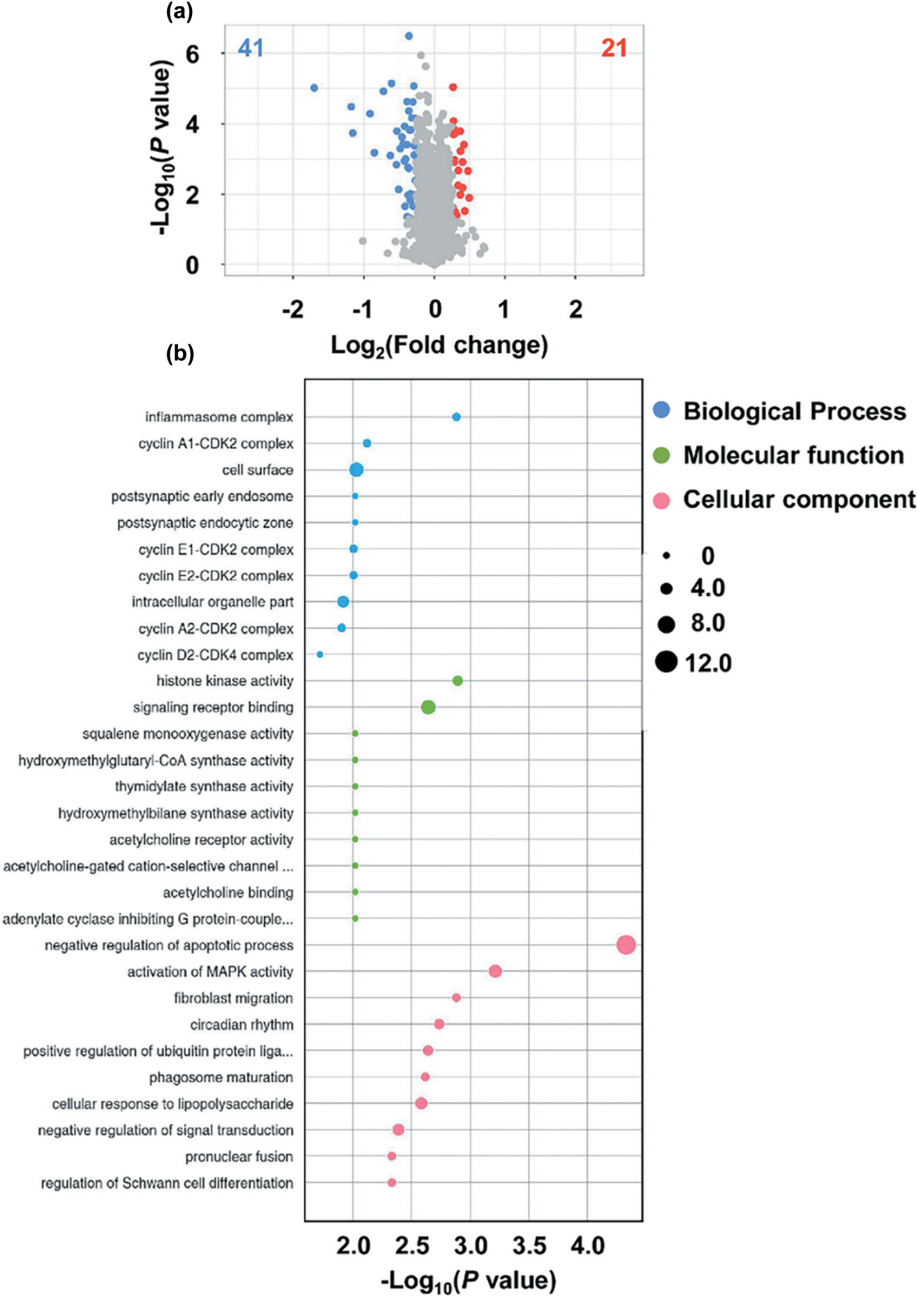
Comparative profiling of the proteomes after downregulation of RBM17. (a) Volcano plots of the proteomes in LV-shRBM17 vs LV-scramble. (b) Bubble chart displaying GO terms for biological process and cellular components. GO terms were functionally grouped depending on Kappa scores and only the most significant GO terms were displayed. The significance of each GO term was displayed as −log10 (*P*-value).

LC–MS analysis was performed to explore RBM17-regulated proteins. Expression alterations in the proteomes were analyzed on RBM17 downregulation in FaDu cells (Figure 5a). Comparative proteome analysis was administrated on three samples per group, and these differentially expressed proteins were identified with ＞1.2-fold changes and *P*-values ＜0.05. There were 62 differentially expressed proteins, 21 upregulated and 41 downregulated. To understand the significant biological function and pathways associated with RBM17, GO enrichment analysis was carried out for biological process, molecular function, and cellular components (Figure 5b). Biological process category analysis showed that these proteins with enhanced localization in the cell surface were associated with cell cycle regulation. Molecular function category analysis demonstrated that these differential proteins of cell membrane were involved in kinase activity and signaling receptor binding. The cellular component category indicated that these differential proteins of cell surface were enriched in the negative regulation of apoptotic process.

**Figure 6:**
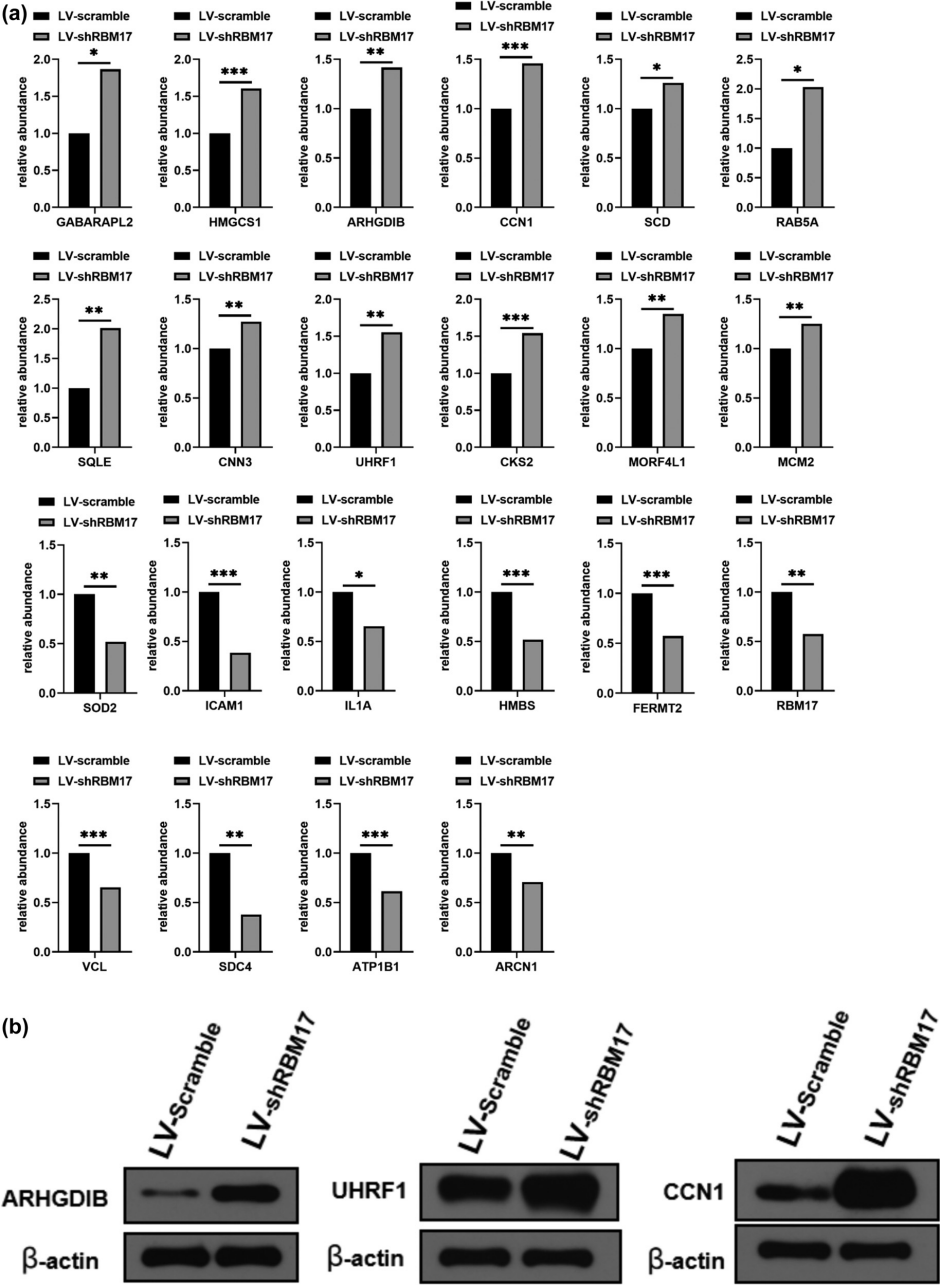
PRM analysis of the differentially expressed proteins. (a) PRM analysis of the differentially expressed proteins after downregulation of RBM17 compared to LV-scramble groups. (b) Selected differentially expressed proteins were confirmed by western blot.

To further verify the abundance of proteins of interest, parallel reaction monitoring (PRM) was utilized for label-free quantification by an MS. Due to the limitation of proteins’ characteristics and their expression abundance, 28 potential proteins were further independently validated by PRM analysis. More than two unique peptides were used for quantifying most protein; however, some proteins were identified only by one peptide because of sensitivity and other reasons. As shown in Figure 6a, 12 proteins (GABARAPL2, HMGCS1, ARHGDIB, CCN1, SCD, SQLE, CNN3, CKS2, RAB5A, MCM2, MORF4L1, and UHRF1) were enriched in RBM17-dowregutated cells, and 9 proteins (HMBS, FERMT2, VCL, SDC4, ATP1B1, ARCN1, SOD2, IL1A, ICAM1) were reduced in RBM17-downregulated cells.

We further confirmed the expression results of three representative proteins by western blot (Figure 6b). Compared with lv-scramble cells, the expressions of ARHGDIB, UHRF1 and CCN1 in LV-shRBM17 cells were increased, which was consistent with the results of PRM.

## 4 Discussion

The pathogenesis and drug resistance of HSCC have complex mechanisms, which are not fully understood at present, and abnormal gene expression may be an important factor. In our previous studies, we found that knockdown of RBM17 can inhibit the proliferation of cancer cells by inducing FaDu cell cycle arrest and apoptosis using flow cytometry [12]. On this basis, this study revealed for the first time the functional role of RBM17 in the regulation of cisplatin sensitivity, migration, invasion, and EMT of hypopharyngeal cancer cells and its downstream-related molecular mechanisms.

We first analyzed the difference of RBM17 expression between tumor and normal tissues using GEPIA and CPTAC databases. The results showed that the expression of RBM17 was significantly increased in HNSCC tumor tissues, and the prognosis of HNSCC patients with high expression of RBM17 was poor. The expression of RBM17 in hypopharyngeal cancer tissues was verified by qRT-PCR and western blot, and it was found that RBM17 was highly expressed in hypopharyngeal cancer tissues. Studies have shown that high expression of RBM17 in hematological tumors can promote the progression of leukemia and may be related to drug resistance of leukemia [18]. Therefore, we speculate that RBM17 may play an important role in chemotherapy resistance of HSCC. It was found that FaDu cells treated with IC_50_ concentration of cisplatin could effectively improve the expression level of RBM17 in mRNA, and knockdown of RBM17 in FaDu cells could enhance the sensitivity of cancer cells to cisplatin. Consistent with our findings, Perry et al. found that RBM17 promotes multidrug resistance in ovarian cancer cells [19]. Through a series of phenotypic experiments, we found that knockdown of RBM17 inhibited the migration and invasion of cancer cells. Li et al. and Lu et al. found that RBM17 had a cancer-promoting effect in liver cancer cells and glioma cells, which was similar to our findings [4,10]. However, different from this study, they mainly studied the regulation of RBM17 on cancer cell cycle and apoptosis and did not involve in the migration and invasion of cancer cells. Our study made up for the deficiency in this aspect.

EMT is a process of epithelial marker loss and mesenchymal phenotype acquisition, and more and more studies have shown that EMT is a key factor leading to tumor cell invasion and metastasis [20]. For example, Liu et al. found that RBM24 could regulate the EMT process of FaDu cells, thus affecting the migration and invasion of hypopharyngeal cancer [21]. In addition, previous studies have shown that EMT plays an important role in tumor cisplatin resistance [22,23,24,25,26]. Our previous studies have shown that RBM17 can affect the migration, invasion ability, and cisplatin sensitivity of hypopharyngeal cancer cells. However, whether the EMT process of hypopharyngeal cancer cells is related to RBM17 has not been reported in the previous literature. To further investigate the effect of RBM17 on FaDu cells, we detected EMT-related markers by qRT-PCR and western blot. Compared with the control group, the expression levels of Vimentin and ZEB1 in LV-shRBM17 cells were significantly decreased. However, the expression level of E-cadherin, an epithelial cell marker, was significantly increased. This suggests that knockdown of RBM17 can inhibit EMT progression in hypopharyngeal cancer. In the present study, we also assessed whether downregulation of RBM17 can effectively inhibit tumor growth in FaDu HSCC xenograft mouse models. We found that knockdown of RBM17 significantly slows tumor growth compared with control mice.

To reveal the potential proteins that were regulated by RBM17 and involved in EMT, herein, the proteomes were comparatively profiled using LC–MS/MS quantitative analysis. Compared with the LV-scramble cells, the experimental group revealed a large number of differential proteins in cell membrane and cytosol. Bioinformatics analysis showed that these differential proteins were significantly enriched during cell cycle regulation and negative regulation of apoptosis and were primarily related to NF-kB signaling and biosynthesis of secondary metabolite pathways. We further screened out 21 differentially expressed proteins by PRM and then selected three representative proteins, ARHGDIB, UHRF1 and CCN1, for western blot verification. Previous study reported that ARHGDIB, also known as RhoGDI2, is a guanosine diphosphate dissociation inhibitor that can decrease cell migration and invasion in bladder cancer [27]. Kim et al. demonstrated that downregulation of UHRF1 contributes to the induction of the EMT in hepatocellular carcinoma cells. UHRF1 deficiency can enhance the migratory and invasive properties of cells via inducing EMT, increasing the tumorigenic capacity of cells and leading to the expansion of cancer stem-like cells [28]. Johnson et al. found that recombinant CCN1 directly inhibited *in vitro* growth of MM cells, and overexpression of CYR61 in MM cells reduced tumor growth [29]. Chen et al. reported that CCN1 suppresses hepatocarcinogenesis by inhibiting carcinogen-induced compensatory hepatocyte proliferation, thus limiting the expansion of damaged and potentially oncogenic hepatocytes [30]. However, the precise mechanisms how RBM17 regulates cisplatin sensitivity and cell invasion in human HSCC FaDu cells through these screened proteins need to be elucidated in the future. In addition, we reviewed the literature and obtained several biomarkers that may be associated with the prognosis of hypopharyngeal cancer, such as PD-L1, SSR1, S100A4, PCDH20, and α2δ1 [31,32,33,34,35]. These biomarkers were compared with the screened differential proteins to try to find out the association between RBM17 and these biomarkers. Unfortunately, we have not been able to prove the potential association between RBM17 and these biological targets based on the data we have so far. More work and experiments are needed subsequently to further reveal the mechanism of action of RBM17 in hypopharyngeal cancer.

In summary, our findings prove that RBM17 is highly expressed in hypopharyngeal cancer and is associated with poor prognosis. Knockdown of RBM17 inhibits FaDu cells *in vitro* and *in vivo* and improves cisplatin sensitivity. More importantly, we observed that downregulation of RBM17 can reverse EMT, and the mechanism may be related to the proteins we screened. Therefore, RBM17 may be a potential therapeutic target for HSCC.

## Abbreviations

CPTAC: clinical proteomic tumor analysis consortium
DFS: disease-free survival
EMT: epithelial–mesenchymal transition
GEPIA: Gene Expression Profiling Interactive Analysis
GTEx: Genotype-Tissue Expression
HNSCC: head and neck squamous cell carcinoma
HSCC: hypopharyngeal squamous cell carcinoma
LC–MS: liquid chromatography–mass spectrometry
MS: mass spectrometry
OS: overall survival
PRM: parallel reaction monitoring
RBM17: RNA-binding motif protein 17
TCGA: The Cancer Genome Atlas
